# Selective Functional Movement Assessment (SFMA) Reliability and Proposal of Its Use in Sports

**DOI:** 10.3390/ijerph20032032

**Published:** 2023-01-22

**Authors:** Andrea Aghi, Daniele Salvagnini, Giovanni Berton, Mattia Cecconi, Elisabetta Della Valle, Rocco Spera, Maria Mambelli, Stefano Palermi, Daniel Neunhaeuserer, Marco Vecchiato

**Affiliations:** 1 Clinica Medica 1, Department of Medicine, University of Padova, 35128 Padova, Italy; 2 Rehabilitation Center “Fisioterapia Raimondi di Giovanni e Daniele”, 35030 Selvazzano Dentro-Padova, Italy; 3 Rehabilitation Center “Daniele Salvagnini”, 35129 Padova, Italy; 4 Rehabilitation Center “Fisiomedic”, Oriago, 30034 Venice, Italy; 5 Public Health Department, University of Naples Federico II, 80131 Naples, Italy; 6 Sports and Exercise Medicine Division, Department of Medicine, University of Padova, 35128 Padova, Italy

**Keywords:** selective functional movement assessment, method assessment, physiotherapists, reliability

## Abstract

Introduction: The Selective Functional Movement Assessment (SFMA) is a functional movement assessment method to observe movement restrictions in individuals with known musculoskeletal disorders, although it has also been used to evaluate healthy athletes of different sports. Aim: The present paper aimed to evaluate the applicability of SFMA in a clinical setting and to verify whether a student can correctly perform it. Methods: An introductory and explanatory email was sent to the subjects, containing the instructions needed to produce a video with SFMA evaluation movements. SFMA methodology was then used to analyze the received videos. The results between interobserver and intraobserver agreement were compared to the literature, considered the gold standard methods. Results: Twenty-eight subjects (17.71 ± 1.96 years aged) were rated. The functional non-painful scenario (FN) has been assigned more frequently by all raters. The student’s intra-rater reliability proved to be moderate (Kappa coefficient 0.49). Results for inter-rater reliability showed that the reliability degree between the senior physiotherapist and student before and after their educational path is good (Kappa coefficient 0.60 and 0.62, respectively). Conclusions: The results of this study showed SFMA intra-rater reliability to be moderate, while inter-rater reliability can be considered good. These characteristics make it a valuable tool for sport’s needs, even when used by students.

## 1. Introduction

The Selective Functional Movement Assessment (SFMA) is a functional movement assessment method devised by Cook and colleagues [1,2] and one of the many tools used by healthcare professionals to observe movement restrictions in individuals with known musculoskeletal disorders [3]. SFMA provides fitness and sports professionals with a logical and reliable method for identifying asymmetries, limitations, imbalances and weaknesses in healthy subjects [1,2].

Recently, a paradigm shift to expand on the biomedical model and include other factors or regions that may contribute to the individuals’ complaints has occurred [4]. Regional interdependence (RI) refers to the concept that seemingly unrelated impairments in a remote anatomical region may contribute to, or be associated with, the patient’s primary complaint [5]. Several authors demonstrated that interventions focused on the thoracic spine can affect cervical impairments [6,7,8] and alter shoulder symptoms [9,10,11]. Deficits in hip strength and abnormal hip mechanics are positively correlated with knee pain in several studies [12,13,14,15]. These studies provide construct validity to the concept of regional interdependence. The RI model presents an opportunity for clinicians to identify remote dysfunctions that may be affecting or causing the patient’s chief complaint, which is an integral role in the SFMA [16]. 

Concerning reliability, three studies show good levels of inter- and intra-rater reliability, showing that SFMA can be consistently scored amongst raters [3,17,18]. 

The SFMA has also been used to evaluate athletes in various sports such as soccer, weightlifting, running and baseball [19,20,21]. In particular, Fauntroy and colleagues analyzed whether the SFMA can be used as a tool to identify the dysfunctions and functional limits of dancers, showing that it contributes to their global functional evaluation [4].

Pre-participation sport screening evaluation methods have been shown to be effective in scientific literature, especially in sport injuries prevention [22,23]. In particular, Palermi et al. showed how a quick musculoskeletal evaluation could have a pivotal role in the screening of athletes, highlighting potentially harmful musculoskeletal abnormal findings. SFMA could, therefore, play a major role in this context, since it represents a rapid and simple tool to be used even by low-experienced physiotherapists [24]. However, to the best of the authors’ knowledge, there are no studies in current literature exploring the role of physiotherapy students in performing such an examination.

Therefore, the aims of this work are the following:

- Analyze SFMA applicability for a physiotherapist in a sport setting.
- Evaluate SFMA reliability for unexperienced raters, once properly trained.

## 2. Materials and Methods

### 2.1. Methodology 

Due to the COVID-19 pandemic, data collection was performed electronically, through video analysis. Subjects were asked to voluntarily participate in the studio, and written informed consent was obtained via email. The study was carried out in accordance with the principles of the Declaration of Helsinki and its later amendments. An introductory and explanatory email was sent to the subjects, containing the instructions needed to produce a video with SFMA evaluation movements. The SFMA methodology was then used to analyze the received videos.

### 2.2. Participants

Study participants were identified as apparently healthy young athletes. As proposed by McKinney and colleagues, “competitive” athletes exercise more than 6 h/week with an emphasis on improving performance, then a minimum of four training sessions per week to achieve at least six cumulative hours per week and five years’ experience in the individual sport were considered [25].

The inclusion criteria were:

- Aged between 15 and 22 years.
- Competitive participation in one of the following sports: football, volleyball, athletics, fencing.
- A minimum of 5 years’ experience in the same sport.
- A frequency of at least 4 training sessions per week.

Exclusion criteria were:

- Previous major musculoskeletal surgery.
- Limb fracture in the last 6 months.
- Limb muscle injury in the last 3 months.

**Figure 1:**
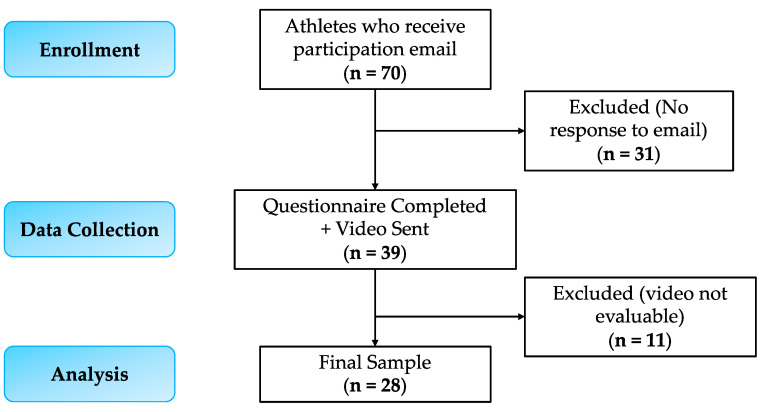
Flowchart diagram for study participants.

The introductory email was sent to 70 athletes who met the inclusion criteria. Only 39 out of 70 of these athletes sent the video containing the performance of the SFMA movements. Moreover, during the video evaluation, an additional 11 athletes were discarded since the movements and/or the video were not performed following the correct instructions. Therefore, our sample size was composed of 28 participants (Figure 1). 

### 2.3. SFMA Evaluation

The athletes were subjected to SFMA evaluation. This evaluation consists of ten movement models which involve all the anatomical regions of the body through the utilization of seven top-tier assessments: cervical patterns—flexion, extension and rotation (right [R] + left [L]); upper-extremity patterns: medial rotation–extension pattern (R + L) and lateral rotation–abduction pattern (R + L); multisegmental patterns: multisegmental flexion; multisegmental extension; multisegmental rotation (R + L); single-leg stance 1 and 2 (with open and closed eyes) (R + L); and overhead squat [26] (Supplementary Table S1). The motor behavior of the patient is investigated in its entirety, creating a movement map divided into: functional non-painful (FN), functional painful (FP), dysfunctional painful (DP) and dysfunctional non-painful (DN). 

The evaluation was performed by a physiotherapist who achieved a “SFMA level 1” certification (rater A) and by a soon-to-be graduated physiotherapy student (rater B1) who underwent a short 8 h training period to learn SFMA. The assessments were conducted separately without interactions between raters. After 60 days the videos were re-examined by rater B1, without access to preceding analysis, after having continued their SFMA educational path (now called rater B2). Data collection was performed between May and August 2020. A different researcher, not involved in the rating processes, performed the statistical analysis and elaboration. 

Glaws and colleagues’ study was taken as a model in comparison with current literature to evaluate inter and intra-SFMA rater reliability [18]. This choice was made because in that study only healthy participants were admitted, rated through video recordings by three raters with different levels of formation: a SFMA instructor with 100 h of formation (rater A’) and two so-called beginner raters with 25 and 8 h of education, respectively (raters B’ and C’) [18]. As a result, according to the level of SFMA education, rater B was compared to rater C’.

### 2.4. Statistical Analysis

The SFMA harvested data were inserted into Microsoft Excel 2019 (Microsoft, Redmond, VA). The data were subsequentially exported in SPSS version 25 (IBM Corporation, Armank, NY) for statistical analysis [27]. Descriptive data are presented as mean and standard deviation for quantitative variables. Discrete variables are presented through absolute and percentage frequency. Analysis to establish SFMA reliability was conducted calculating absolute agreement and Cohen’s Kappa coefficient (K). Confidence intervals (CI) at 95% for K coefficient were calculated using Mc Hugh’s recommended formula: (K ± 1.96 × SEk (Standard error)) [28]. The statistical analysis to define inter and intra-rater SFMA reliability was conducted using the aforementioned calculation of the Kappa coefficient [29]. A minimum sample size of 24 athletes would be sufficient for this study to achieve a Cohen’s kappa value of 0.50 with an alpha of 0.05 and a power of 80% for a two-tailed test [30]. For the definition of the degree of concordance obtained from the value of Kappa, the classification proposed by Landis and Koch is used: k < 0, no agreement; 0 ≤ k < 0,4 slight/fair concordance; 0.4 ≤ k < 0.6 moderate concordance; 0.6 ≤ k < 0.8 substantial/good concordance; 0.8 ≤ k ≤ 1 great concordance [31].

## 3. Results

The included participants were mainly male and half of them played soccer (Table 1).

Supplementary Table S2 shows the movement map assigned by rater A, rater B1 and rater B2. The FN scenario was assigned more frequently to the following movements than the others by all raters: single leg position for the left and right leg (SLS right and left) and superior limb pattern for the left limb (UE 2 left). The dysfunctional non-painful (DN) score was assigned with more frequency to the following patterns: cervical flexion (C Flex), multi-segmental flexion (MSF), complete squat (OHDS) and superior limb pattern 1 for the right limb (UE 1 right). The highest number of painful movements, later evaluated as dysfunctional or functional (FP or DP), was found in the following patterns: superior limb pattern 1 for the right limb (UE 1 right), multi-segmental extension (MSE) and complete squat (OHDS). Overall, the DN pattern was assigned a higher number of times by rater A, followed by rater B2 and B1, while there is a similar percentage in the assignation of pattern in the presence of pain (FP or DP).

Table 2 reports the results for rater B intra-rater reliability, which proved to be moderate (0.49). The highest Kappa value is found in the following movements: multi segmental flexion (0.88) and multi segmental extension (0.74); whereas the lowest K value is found in the following patterns: superior limb pattern 2 for the right limb (0.16) and left limb (0.29), and in the single leg closed eye (Single Leg Stance 2) for the right limb position (0.22). The single leg open eyes for the left limb position (Single Leg Stance 1 left) does not have a Kappa coefficient since rater B2 assigned an FN valuation to all the participants. Thus, the variable was considered as constant by the software and the coefficient was not calculated. Table 2 also presents a comparison between rater B and C’ intra-rater reliability. The Kappa value associated with evaluator C’ intra-rater reliability (0.62) is higher than the same value for rater B (0.49).

Results for inter-rater reliability are described in Table 3, showing that the reliability degree for rater A and B1 and A and B2 is good (0.60 and 0.62, respectively), with a slight improvement between rater A and B2. The main improvements between A and B2, compared to A and B1, are found in the following movements: left cervical rotation (0.47→0.80), where there is an improvement from decent to great, left multi-segmental rotation (0.38→0.74) and complete squat (0.38→0.74), where there is an improvement from poor to good. The most notable worsening in Kappa value is found in the following movements: superior limb pattern 1 for the left limb (0.61→0.30) and single leg closed eyes for the right limb position (0.53→0.32). In the mean between A and B1 and A and B2, the only poor movement is superior limb pattern 1 for the left limb (0.39), despite a marked improvement in Kappa value (0.22→0.57).

Table 3 also presents the comparison between raters A and B1 inter-rater reliability and raters A’ and C’ inter-rater reliability. Reliability is higher between raters A and B1 (0.60). The only movement where the Kappa value is higher between A’ and C’ is the superior limb pattern 1 for the left limb; however, the difference is minimal and concordance is low in both cases (Kappa A vs. B1 = 0.22; Kappa A’ vs. C’ = 0.28). Both tables lack Kappa coefficients for the single leg closed eyes position (SLS 2 right and SLS 2 left) since in Glaws and colleagues’ study those movements were not taken into consideration during the evaluation [18].

## 4. Discussion

SFMA is an evaluation model of functional movement for physicians and healthcare professionals to help individuate causes of pain due to musculoskeletal problems [32]. This observational study aimed to analyze SFMA reliability when used by a physiotherapy student with a short period of formation. Pre-existing studies assert that SFMA presents adequate reliability when performed by experienced raters [3,17,18]. Despite low experience and limited SFMA training, the results showed that inter and intra-rater reliability degree for the student ranges from moderate to good (0.49 and 0.62, respectively).

Glaws and colleagues’ article was taken as a model for the methodological consistency of this study, but it is not the only one present in the literature that tried to evaluate inter and intra-rater SFMA reliability. In 2019, Stanek organized a study with the same focus, although evaluations were conducted live by three raters with varying degrees of experience [3,18]. Despite these differences between live and video evaluation, the rater B inter-rater reliability degree (0.49) is comparable to all three raters from Stanek’s study (0.55, 0.50, 0.48). Moreover, the inter-rater reliabilities between A and B1 (0.60) and between A and B2 (0.62) are higher than the compared study (0.54, 0.47, 0.44), and even higher than the expert rater in this study [3]. 

Our results show that some movements have a higher degree of reliability compared to others. Indeed, when considering rater B intra-rater reliability, Kappa value rises from 0.16 to 0.88, while inter-rater reliability results rise from 0.22 to 1. This can be explained because some movements have evaluation criteria that are more objective than others. Cervical flexion, multi-segmental flexion and extension are the movements with higher intra and inter-rater reliability levels, while movement models rating the two limbs separately (superior limb and single leg closed eyes pattern) and complete squat movements have demonstrated a lower reliability. This is in line with what is reported in the literature, since these aforementioned movements, despite having objective criteria, present subjective elements to be rated: quality and motor control of the movement and dominance [3]. 

Reliability study is just the first element to be analyzed to define relevance in the clinical practice of a tool. Bannigan and Watson in 2009 stated that to establish the usefulness of an evaluation tool, it is necessary to define in which measure said tool is reliable, valid and usable [33]. To date, SFMA evaluation validity has been investigated by only one study [34]. Busch and colleagues utilized SFMA in their 2017 study at the beginning of the sports season on sixty baseball players noting that altered movements to the evaluation were correlated to higher probabilities of feeling painful overload symptoms both during pre-season and season periods [21]. Moreover, in a revision study to define functional limitations and dysfunctions in dancers, Fauntroy asserted that SFMA as functional movement screening is useful and relevant for improving clinical evaluation and to identify motorial dysfunction in both these subjects and in athletes from other team sports to help reduce the incidence of lesions [4].

SFMA was also used to prescribe a tailored exercise program, after evaluating patients’ mobility and stability complaints [35]. This is an interesting aspect to be considered in the field of sport medicine, when prescribing a customized physical activity in patients [36] to be performed in specific medical gyms [37]. A recent study proposes the Arm Care Screen (ACS) as a new tool based on the concepts of the SFMA in high school coaches and investigated intra-rater and inter-rater reliability when administered by high school baseball coaches in a sample of baseball players. The ACS provides coaches with a tool to track changes in physical function throughout the season so that exercise intervention and/or further evaluation can be recommended prior to the onset of injury [38]. Only two studies (a case report and a case series) highlighted the usefulness of SFMA in the evaluation of painful athletes underlying how SFMA helps identifying problems related to mobility and stability in certain specific patterns of movement [35,39]. 

Unfortunately, our study suffers from some limitations. Firstly, our sample size was small and made up of nearly all male subjects. Furthermore, due to the COVID-19 pandemic, the assessments were carried out through recordings, and it was only possible to monitor the professional athletes of four competitive sports; however, this highlights the potential use of SFMA assessment even when not performed in person. Despite these limitations, this study provided positive feedback on the evaluation of SFMA and can be considered a first step towards developing future studies on the possible role of SFMA in preventive evaluation in sports physiotherapy. Showing how even a physiotherapy student can examine acceptably, this study highlights how SFMA represents a simple but effective tool both to be performed and taught.

## 5. Conclusions

SFMA is a quick functional movement assessment tool with intuitive, simple and clear language. The results of this study showed that SFMA intra-rater reliability is moderate, while SFMA inter-rater reliability can be considered good. These characteristics make it a valuable tool for sports, even when used by students. Therefore, we suggest the need for further future studies to analyze SFMA in sports practice and the individuation of motor dysfunctions in athletes.

## Figures and Tables

**Table 1:** Characteristics of the sample size (n = 28).

Variable	
Age (years)—mean, SD	17.71 ± 1.96
Male, n (%)	25 (89.3%)
Sports activity—n (%)	Athletics—6 (21.4%) Soccer—14 (50%) Volleyball—5 (17.9%) Fencing—3 (10.7%)
Years of practice—mean, SD	9.82 ± 2.37

SD: Standard Deviation.

**Table 2:** Intra-rater reliability of rater B.

	Intra-Rater B		Intra-Rater C’	
Intra-Rater Comparison: Rater B and Rater C (Glaws and Colleagues)	Kappa	% Agreement	Kappa	% Agreement
C Flex	0.654 (0.380–0.928)	0.821	0.440	0.890
C Ext	0.517 (0.198–0.836)	0.821	0.800	0.940
C Rot R	0.633 (0.355–0.911)	0.821	0.600	0.890
C Rot L	0.426 (0.140–0.712)	0.714	0.530	0.860
UE pattern 1 Right	0.397 (0.074–0.720)	0.643	0.660	0.830
UE pattern 1 Left	0.397 (0.027–0.767)	0.750	0.610	0.830
UE pattern 2 Right	0.164 (−0.275–0.603)	0.786	0.600	0.890
UE pattern 2 Left	0.294 (−0.106–0.694)	0.786	0.410	0.830
MSF	0.877 (0.710–1.044)	0.926	0.720	0.860
MSE	0.744 (0.530–0.958)	0.852	0.250	0.630
MSR Right	0.584 (0.214–0.954)	0.893	0.770	0.890
MSR Left	0.462 (0.176–0.748)	0.750	0.620	0.830
SLS 1 Right	0.481 (−0.111–1.073)	0.929	0.760	0.890
SLS 1 Left	/		0.830	0.910
SLS 2 Right	0.22 (−0.005–0.445)	0.607	/	
SLS 2 Left	0.5 (0.122–0.878)	0.821	/	
OHDS	0.541 (0.306–0.776)	0.714	0.720	0.910
Mean	0.493	0.790	0.621	0.859
SD	0.185	0.092	0.161	0.072

C Flex: Cervical Flexion; C Ext: Cervical Extension; C Rot R: Cervical Rotation Right; C Rot L: Cervical Rotation Left; UE pattern 1 Right: Upper Extremity Pattern 1 Right; UE pattern 1 Left: Upper Extremity Pattern 1 Left; UE pattern 2 Right: Upper Extremity Pattern 2 Right; UE pattern 2 Left: Upper Extremity Pattern 2 Left; MSF: Multi-Segmental Flexion; MSE: Multi-Segmental Extension; MSR Right: Multi-Segmental Rotation Right; MSR Left: Multi-Segmental Rotation Left; SLS 1 Right: Single Leg Stance 1 Right; SLS 1 Left: Single Leg Stance 1 Left; SLS 2 Right: Single Leg Stance 2 Right; SLS 2 Left: Single Leg Stance 2 Left; SD: Standard Deviation.

**Table 3:** Inter-rater reliability (evaluator A vs. B1 and A vs. B2).

Inter-Rater Reliability Rater B	Rater A vs. B1		Rater A vs. B2		Inter-Rater A’ vs. C’	
	Kappa	% Agreement	Kappa	% Agreement	Kappa	% Agreement
C Flex	0.852 (0.652–1.052)	0.929	0.793 (0.573–1.013)	0.893	0.30	0.74
C Ext	0.687 (0.362–1.012)	0.893	0.663 (0.379–0.947)	0.857	0.62	0.86
C Rot R	0.478 (0.149–0.807)	0.786	0.494 (0.204–0.784)	0.75	0.30	0.83
C Rot L	0.472 (0.174–0.770)	0.75	0.796 (0.575–1.017)	0.893	0.32	0.74
UE pattern 1 Right	0.588 (0.321–0.855)	0.75	0.462 (0.168–0.756)	0.679	0.19	0.60
UE pattern 1 Left	0.218 (−0.168–0.604)	0.643	0.569 (0.236–0.902)	0.821	0.28	0.63
UE pattern 2 Right	0.531 (0.066–0.996)	0.893	0.525 (0.051–0.999)	0.893	0.53	0.86
UE pattern 2 Left	0.611 (0.229–0.993)	0.893	0.3 (−0.104–0.704)	0.75	0.58	0.86
MSF	0.813 (0.607–1.019)	0.889	0.816 (0.618–1.014)	0.889	0.44	0.71
MSE	0.681 (0.430–0.932)	0.815	0.806 (0.604–1.008)	0.889	0.27	0.66
MSR Right	0.517 (0.198–0.836)	0.821	0.614 (0.281–0.947)	0.857	0.33	0.66
MSR Left	0.376 (0.111–0.641)	0.679	0.737 (0.494–0.980)	0.857	0.34	0.77
SLS 1 Right	0.786 (0.382–1.190)	0.964	0.654 (0.033–1.275)	0.964	0.61	0.80
SLS 1 Left	1	1	/		0.60	0.80
SLS 2 Right	0.528 (0.220–0.836)	0.75	0.319 (0.027–0.611)	0.714	/	
SLS 2 Left	0.7 (0.384–1.016)	0.893	0.576 (0.204–0.948)	0.857	/	
OHDS	0.309 (0.070–0.548)	0.536	0.726 (0.501–0.951)	0.821	0.18	0.83
Mean	0.5969	0.8167	0.6156	0.8365	0.39	0.76
SD	0.20	0.122	0.17	0.077	0.156	0.088

C Flex: Cervical Flexion; C Ext: Cervical Extension; C Rot R: Cervical Rotation Right; C Rot L: Cervical Rotation Left; UE pattern 1 Right: Upper Extremity Pattern 1 Right; UE pattern 1 Left: Upper Extremity Pattern 1 Left; UE pattern 2 Right: Upper Extremity Pattern 2 Right; UE pattern 2 Left: Upper Extremity Pattern 2 Left; MSF: Multi-Segmental Flexion; MSE: Multi-Segmental Extension; MSR Right: Multi-Segmental Rotation Right; MSR Left: Multi-Segmental Rotation Left; SLS 1 Right: Single Leg Stance 1 Right; SLS 1 Left: Single Leg Stance 1 Left; SLS 2 Right: Single Leg Stance 2 Right; SLS 2 Left: Single Leg Stance 2 Left, SD: Standard Deviation.

## Data Availability

The data that support the findings of this study are available from the first author, A.A., upon reasonable request.

## Supplementary Materials

The following supporting information can be downloaded at: https://www.mdpi.com/article/10.3390/ijerph20032032/s1, Table S1. SFMA instructions. Table S2. SFMA movement map assigned by Rater A, Rater B1 and Rater B2.

## Abbreviation

SFMA	Selective Functional Movement Assessment;
FN	Functional non-painful;
FP	Functional painful;
DN	Dysfunctional non-painful;
DP	Dysfunctional painful;
RI	Regional interdependence
C Flex	Cervical Flexion;
C Ext	Cervical Extension;
C Rot R	Cervical Rotation Right;
C Rot L	Cervical Rotation Left;
UE pattern 1 Right	Upper Extremity Pattern 1 Right;
UE pattern 1 Left	Upper Extremity Pattern 1 Left;
UE pattern 2 Right	Upper Extremity Pattern 2 Right;
UE pattern 2 Left	Upper Extremity Pattern 2 Left;
MSF	Multi-Segmental Flexion;
MSE	Multi-Segmental Extension;
MSR Right	Multi-Segmental Rotation Right;
MSR Left	Multi-Segmental Rotation Left;
SLS 1 Right	Single Leg Stance 1 Right;
SLS 1 Left	Single Leg Stance 1 Left;
SLS 2 Right	Single Leg Stance 2 Right;
SLS 2 Left	Single Leg Stance 2 Left;
K	Cohen’s Kappa
