# Supplementary material for: Selective Functional Movement Assessment (SFMA) Reliability and Proposal of Its Use in Sports
Source: Int J Environ Res Public Health. 2023 Jan 22;20(3):2032. doi: 10.3390/ijerph20032032 (PMC9915240; doi:10.3390/ijerph20032032)
Supplement: Supplementary file 1 [file ijerph-20-02032-s001.zip › supplementary Table S1.pdf]

Supplementary Table S1: SFMA instructions.

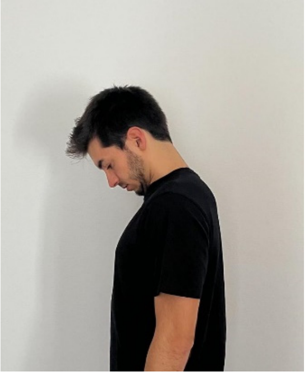

Active Cervical Flexion

Instruction: Stand erect with feet together, toes pointing forward. Bring your chin down to your chest with mouth closed.

Criteria: Chin touches sternum with mouth closed

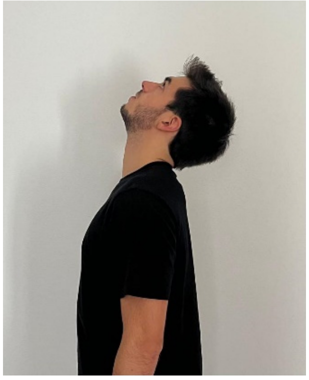

Active Cervical Extension

Instruction: Stand erect with feet together, toes pointing forward. Look up to the ceiling as far as you can.

Criteria: Head reaches > 10° of parallel

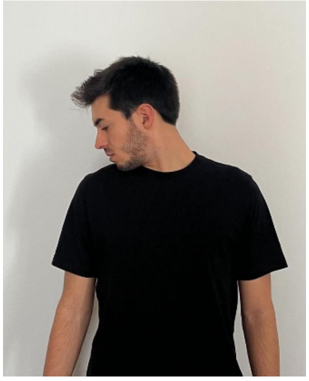

Cervical Rotation Side-Bend

Instruction: Stand erect with feet together, toes pointing forward. Turn your head as far as you can to the right/left and then flex your neck moving chin to collarbone. (Note: Avoid shoulder elevation)

Criteria: Chin touches mid-clavicle

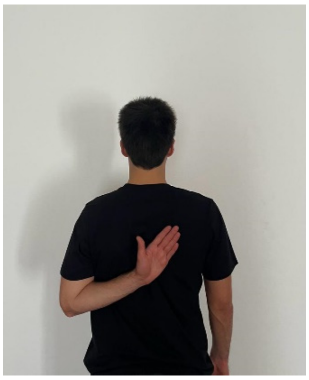

Upper Extremity Pattern 1 – Medial Rotation Extension

Instruction: Stand erect with feet together, toes pointing forward. Take your right/left arm and reach behind your back aiming to touch the bottom of your opposite shoulder blade.

Criteria: Touches inferior angle of contralateral scapula

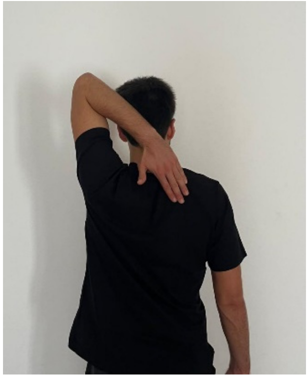

Upper Extremity Pattern 2 – Lateral Rotation Flexion

Instruction: Stand erect with feet together, toes pointing forward. Take your right/left arm and reach up and behind your head aiming to touch the top of your opposite shoulder blade.

Criteria: Touches spine of contralateral scapula

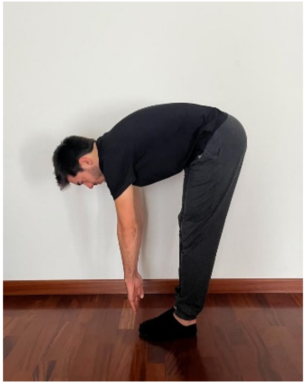

Multi-segmental Flexion

Instruction: Stand erect with feet together and shoes off, toes pointing forward. Bend forward and try to touch your toes. (Note: knees straight)

Criteria: Touches toes and returns to standing position; Sacral angles > 70°; Presence of posterior weight shift (T-L junction over foot); Uniform spinal curves

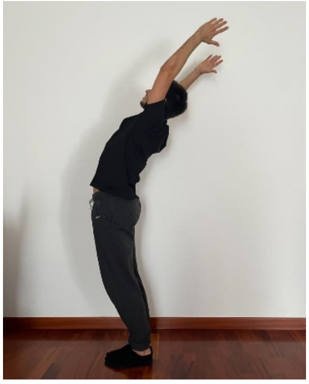

Multi-segmental Extension

Instruction: Stand erect with feet together and shoes off, toes pointing forward. Keep your hand above your head and bend backwards as far as you can. (Note: knees straight)

Criteria: ASIS clears toes; Maintains normal shoulder flexion (≥ 170°); Spine of scapula clears heels; Uniform spinal curves

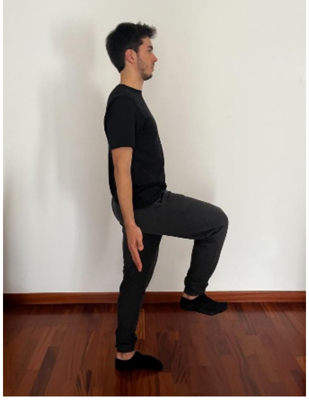

Multi-segmental Rotation

Instruction: Stand erect with feet together and shoes off, toes pointing forward. Place your hand by your side and rotate your entire body right/left as far as you can while keeping your feet still

Criteria: Pelvis rotation ≥ 50°; Trunk/shoulder rotation ≥ 50°; No deviation of spine and pelvis; Limited knee flexion needed to achieve movements

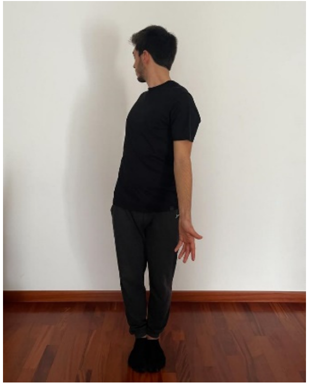

Single Leg Stance (eyes open, then closed)

Instruction: Stand erect with feet together and shoes off, toes pointing forward. Lift you right/left knee to hip and hold for 10 seconds. Then do the same with eyes closed.

Criteria: maintains for 10 seconds (eyes open and closed); no loss of height (bending knee)

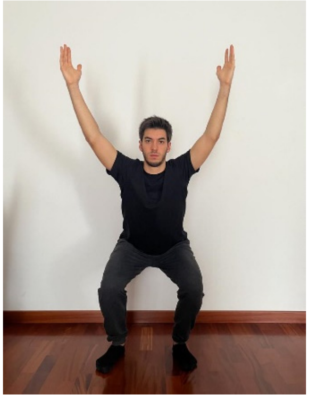

Overhead Deep Squat

Instruction: Stand feet shoulder width apart with shoes off and toes pointing forward. Place your arms over your head. Hold this position and squat down as far as you can towards the floor.

Criteria: Maintains shoulder flexion; Maintains neutral thoracic spine (no flexion); Femur > than parallel to floor; No sagittal plane deviation of lower extremities
