# Supplementary material for: Selective Functional Movement Assessment (SFMA) Reliability and Proposal of Its Use in Sports
Source: Int J Environ Res Public Health. 2023 Jan 22;20(3):2032. doi: 10.3390/ijerph20032032 (PMC9915240; doi:10.3390/ijerph20032032)
Supplement: Supplementary file 1 [file ijerph-20-02032-s001.zip › supplementary Table S2.pdf]

International Journal of
Environmental Research and Public Health

MDPI

Supplementary Table S2. SFMA movement map assigned by Rater A, Rater B1 and Rater B2**.**

	FN: Functional non painful			FP: Functional painful			DN: Dysfunctional non painful			DP: Dysfunctional painful		
	**Rater A**	**Rater B1**	**Rater B2**	**Rater A**	**Rater B1**	**Rater B2**	**Rater A**	**Rater B1**	**Rater B2**	**Rater A**	**Rater B1**	**Rater B2**
**Cervical Flexion: C Flex**	18 (64.3%)	18 (64.3%)	15 (53.6%)	1 (3.6%)	1 (3.6%)	1 (3.6%)	9 (32.1%)	9 (32.1%)	12 (42.9%)	0 (0%)	0 (0%)	0 (0%)
**Cervical Extension: C Ext**	21 (75%)	24 (85.7%)	20 (71.4%)	1 (3.6%)	1 (3.6%)	2 (7.1%)	5 (17.9%)	2 (7.1%)	6 (21.4%)	1 (3.6%)	1 (3.6%)	0 (0%)
**Cervical Rotation Right: C Rot R**	20 (71.4%)	21 (75%)	16 (57.1%)	1 (3.6%)	0 (0%)	0 (0%)	7 (25%)	6 (21.4%)	11 (39.3%)	0 (0%)	1 (3.6%)	1 (3.6%)
**Cervical Rotation Left: C Rot L**	16 (57.1%)	23 (82.1%)	15 (53.6%)	0 (0%)	0 (0%)	0 (0%)	11 (39.3%)	4 (14.3%)	12 (42.9%)	1 (3.6%)	1 (3.6%)	1 (3.6%)
**UE pattern 1 Right**	14 (50%)	14 (50%)	16 (57.1%)	1 (3.6%)	0 (0%)	0 (0%)	10 (35.7%)	10 (35.7%)	8 (28.6%)	3 (10.7%)	4 (14.3%)	4 (14.3%)
**UE pattern 1 Left**	19 (67.9%)	19 (67.9%)	22 (78.6%)	0 (0%)	0 (0%)	0 (0%)	8 (28.6%)	8 (28.6%)	5 (17.9%)	1 (3.6%)	1 (3.6%)	1 (3.6%)
**UE pattern 2 Right**	25 (89.3%)	24 (85.7%)	24 (85.7%)	0 (0%)	1 (3.6%)	0 (0%)	2 (7.1%)	2 (7.1%)	3 (10.7%)	1 (3.6%)	1 (3.6%)	1 (3,6%)
**UE pattern 2 Left**	22 (78.6%)	25 (89.3%)	21 (75%)	0 (0%)	0 (0%)	0 (0%)	6 (21.4%)	3 (10.7%)	7 (25%)	0 (0%)	0 (0%)	0 (0%)
**Multi-Segmental Flexion: MSF**	14 (50%)	13 (46.4%)	11 (39.3%)	1 (3.6%)	1 (3.6%)	1 (3.6%)	10 (35.7%)	11 (39.3%)	13 (46.4%)	2 (7.1%)	2 (7.1%)	2 (7.1%)
**Multi-Segmental Extension: MSE**	16 (57.1%)	16 (57.1%)	16 (57.1%)	4 (14.3%)	3 (10.7%)	5 (17.9%)	6 (21.4%)	6 (21.4%)	6 (21.4%)	1 (3.6%)	2 (7.1%)	0 (0%)
**Multi-Segmental Rotation Right: MSR Right**	20 (71.1%)	24 (85.7%)	24 (85.7%)	1 (3.6%)	2 (7.1%)	2 (7.1%)	6 (21.4%)	2 (7.1%)	2 (7.1%)	1 (3.6%)	0 (0%)	1 (3,6%)
**Multi-Segmental Rotation Left: MSR Left**	15 (53.6%)	23 (82.1%)	17 (60.7%)	2 (7.1%)	1 (3.6%)	2 (7.1%)	11 (39.3%)	3 (10.7%)	9 (32.1%)	0 (0%)	0 (0%)	0 (0%)
**Single Leg Stance 1 Right: SLS 1 Right**	26 (92.9%)	25 (89.3%)	27 (96.4%)	1 (3.6%)	1 (3.6%)	1 (3.6%)	1 (3.6%)	2 (7.1%)	0 (0%)	0 (0%)	0 (0%)	0 (0%)
**Single Leg Stance 1 Left: SLS 1 Left**	27 (96.4%)	27 (96.4%)	28 (100 %)	0 (0%)	0 (0%)	0 (0%)	1 (3.6%)	1 (3.6%)	0 (0%)	0 (0%)	0 (0%)	0 (0%)
**Single Leg Stance 2 Right: SLS 2 Right**	17 (60.7%)	14 (50%)	25 (89.3%)	0 (0%)	0 (0%)	0 (0%)	10 (35.7%)	13 (46.4%)	3 (10.7%)	1 (3.6%)	1 (3.6%)	0 (0%)
**Single Leg Stance 2 Left: SLS 2 Left**	22 (78.6%)	21 (75%)	22 (78.6%)	0 (0%)	0 (0%)	0 (0%)	6 (21.4%)	7 (25%)	6 (21.4%)	0 (0%)	0 (0%)	0 (0%)
**Overhead Deep Squat: OHDS**	10 (35.7%)	18 (64.3%)	13 (46.4%)	2 (7.1%)	3 (10.7%)	2 (7.1%)	13 (46.4%)	5 (17.9%)	10 (35.7 %)	3 (10.7%)	2 (7.1%)	3 (10.7%)
